# Hemophagocytic Lymphohistiocytosis as a Manifestation of Underlying Visceral Leishmaniasis

**DOI:** 10.7759/cureus.11911

**Published:** 2020-12-04

**Authors:** Michael D Diamantidis, Andromachi Palioura, Maria Ioannou, Evangelos Tsangalas, Konstantinos Karakousis

**Affiliations:** 1 Hematology, First Department of Internal Medicine, Thalassemia and Sickle Cell Disease Unit, General Hospital of Larissa, Larissa, GRC; 2 Internal Medicine, First Department of Internal Medicine, General Hospital of Larissa, Larissa, GRC; 3 Pathology, Haemopathology, University Hospital of Larissa, University of Thessaly, Larissa, GRC

**Keywords:** hemophagocytic lymphohistiocytosis (hlh), hemophagocytic syndrome (hs), macrophage activation syndrome (mas), visceral leishmaniasis (vl), liposomal amphotericin b

## Abstract

Hemophagocytic lymphohistiocytosis (HLH), or hemophagocytic syndrome (HS) is a severe syndrome involving an extreme participation of the immune system, resulting in a cascade of cytokines, hyperinflammation and extensive hemophagocytosis in the bone marrow (BM) and affecting the peripheral blood (PB) lineages. Fever, splenomegaly, hypertriglyceridemia, hypofibrinogenemia, and hyperferritinemia are often encountered in this disease. The syndrome can be seen in all ages and it is either primary due to genetic defects or secondary because of malignancies, immune deficiencies, rheumatic diseases, and infections. Bacteria, viruses, protozoa, and fungi are often implicated. Visceral leishmaniasis (VL) is among the infectious causes of HLH. We describe a patient with a successful treatment of HLH after the initiation of liposomal amphotericin B, due to VL, even though there was a delay in diagnosing the leishmaniasis. The exact precipitating pathophysiological events triggering HLH remain unknown and provide their clear impact for future research. An instructive, critical review of the literature related to the presented case is provided. Distinguishing secondary HS from primary HS is essential for the application of suitable treatment. Improper use of corticosteroids could cover up an underlying possible malignancy or infection and delay the initiation of the etiologic therapeutic strategy.

## Introduction

The etymology of the word “hemophagocytosis” comes from the combination of the Greek words haema (=blood), phago (=eat), and cytos (=container). It is used to describe the pathological condition of several types of cells in the bone marrow (BM), usually macrophages, destroying other cells or large particles by engulfing them with their plasma membrane, giving rise to an internal compartment, called the phagosome.

Hemophagocytic lymphohistiocytosis (HLH) is an aggressive and life-threatening syndrome of excessive immune activation observed in children and adults of all ages. It is also called hemophagocytic syndrome (HS) or macrophage activation syndrome (MAS). More precisely, the clinical syndrome caused by HLH should be named HS. Nevertheless, there is often overlap in the literature between the two terms, both referring to the same life-threatening condition.

HLH can occur as a familial or sporadic disorder, and might be triggered by a variety of events that disrupt immune homeostasis. Infection is a common trigger both in those with a genetic predisposition and in sporadic cases. Genetic or primary HLH has been linked to distinct molecular or chromosomal aberrations [1]. Acquired or secondary HLH derives either from exogenous agents, such as infectious organisms or toxins, or from endogenous products, related to tissue damage, radical stress, and metabolic products [2]. Rheumatic diseases, immune deficiencies [1], and malignancies, such as lymphomas or multiple myeloma, can also trigger HLH.

Interestingly, HLH secondary to disseminated histoplasmosis in systemic lupus erythematosus [3] or in patients with human immunodeficiency virus (HIV) [4,5] has been described. Moreover, from the newest immunomodulatory monoclonal antibodies, the drug daratumumab has been implicated for inducing HLH as well [6].

Visceral leishmaniasis (VL) is among the infectious causes of HLH. The diagnosis can sometimes be tricky. We describe a case of HLH as a manifestation of VL.

## Case presentation

An 82-year-old female with arterial hypertension controlled with ramipril presented to our center with fever (38.6°C) for four days without chills. She reported mild weakness with unclear onset and lacked other B-symptoms, such as night sweats or weight loss. Laboratory results showed pancytopenia (white blood cells (WBCs) 1,600/μL, neutrophils 970/μL, lymphocytes 580/μL, monocytes 40/μL, hemoglobin 10.7 g/dL, platelets 85,000/μL). She was admitted to the Internal Medicine Department for further evaluation and treatment.

Additional diagnostic tests showed increased ferritin (7,146 ng/ml [normal <307 ng/ml]), triglycerides [390 mg/dl (normal <150 mg/dl)] and lactate dehydrogenase (LDH) levels [1,358 U/L (normal <480 U/L)]. Fibrinogen levels were low [134 mg/dl (normal 200-400)]. After two days, fibrinogen further decreased (64 mg/dl), while ferritin improved slightly (5,167 ng/ml), still much above the normal range. Empirical treatment with ampicillin plus tazobactam was initiated, whereas three consequent blood cultures under fever from peripheral blood were negative. A complete infection work-up (Epstein-Barr virus (EBV), cytomegalovirus (CMV), herpes simplex virus (HSV), hepatitis B virus (HBV), hepatitis C virus (HCV), HIV, toxoplasma, Widal, Wright) was also negative. The patient had no splenomegaly. Chest and abdomen CT showed no significant radiological findings or lymphadenopathy. Because of the laboratory data, hemophagocytic syndrome was suspected.

**Figure 1:**
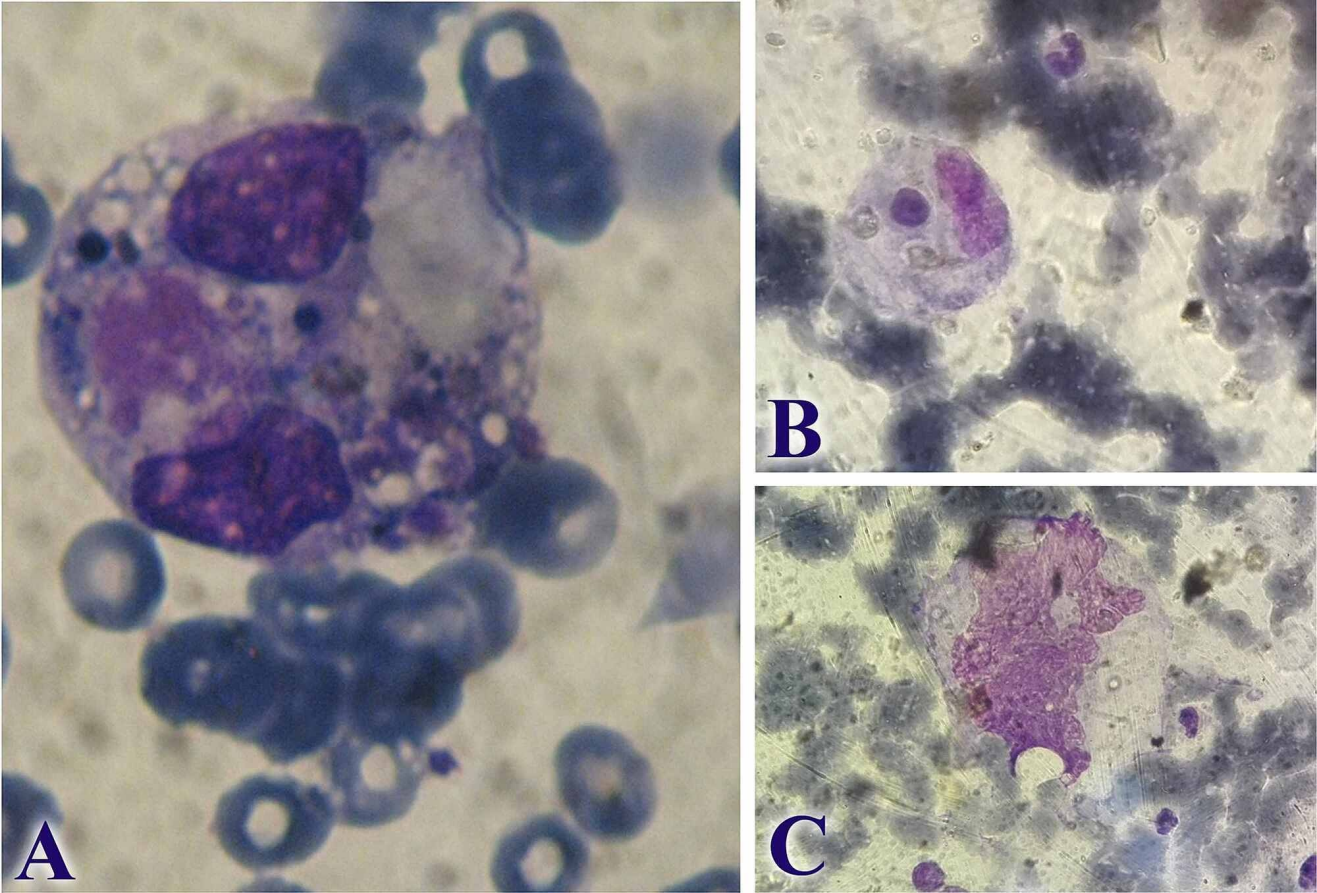
Bone Marrow Aspiration A. Extensive hemophagocytosis by histiocytes of erythroblasts, red cells, platelets and remnants of nuclei resembling monocytes; B. Hemophagocytosis of cellular elements at a lesser degree; C. Hemophagocytosis of a cellular element resembling a remnant of a megakaryocyte.

On day five, bone marrow (BM) aspiration yielded extensive hemophagocytosis by histiocytes of erythroblasts, red cells, platelets, and remnants of nuclei resembling monocytes (Figure 1, A-C). Precursors of all cell lines were also eliminated by the phagocytes (Figure 1, A-C). Parasites resembling leishmanias were observed and an additional screening for leishmaniasis, which involved polymerase chain reaction (PCR), antibodies for leishmanias plus a pending BM biopsy, was recommended.

Thus, five out of eight criteria for HLH (fever, cytopenias involving two lineages, hypertriglyceridemia and hypofibrinogenemia, hemophagocytosis in BM and hyperferritinemia) were fulfilled [7]. Measuring a possible low natural killer (NK) cell activity and the soluble CD25 [interleukin (IL)-2 receptor] levels were not available in our department and were not conducted. Even though the leishmania parasites were observed in the BM aspirate, etiological treatment for the infection was not initiated, due to the pending BM biopsy. On day six, the suppression of the excessive activation of the immune system was proposed as a therapeutic strategy and intravenous prednisolone at the dosage of 25 mg, three times daily, was initiated. As the fever persisted, ampicillin and tazobactam were discontinued and ceftriaxone plus meropenem were applied.

On day 10, antibodies for leishmanias with the technique of immunochromatography were positive and a history of direct contact of the patient with canines was discovered. On day 13, platelets further decreased to 55,000/μL, while on day 16, ferritin levels reached their highest value of 10,500 ng/ml and LDH was 1,873 U/L. Human normal immunoglobulins [300 mg/kg (5 g/100ml)] were applied as an attempt to control the thrombocytopenia, but with no result. A polyclonal increase of immunoglobulin (Ig) A and IgM immunoglobulins was observed, as expected in leishmaniasis. On day 17, platelets were as low as 30,000/μL.

**Figure 2:**
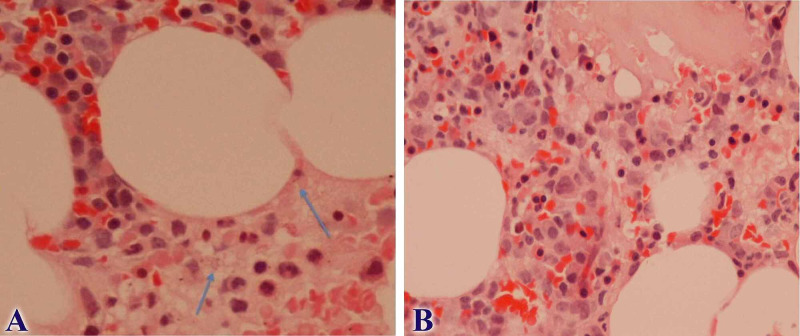
Bone Marrow Biopsy A. Cells with granules, suspect for intracellular parasite of the type of leishmania (blue arrows). Hematoxylin and Eosin (HE) stain X 400; B. Overall imaging of the bone marrow showing leishmanias. Hematoxylin and Eosin (HE) stain X 100

Finally, 19 days after the initial admission of the patient, BM trephine biopsy showed cells with granules, suspect for intracellular parasite of the type of leishmania (Figure 2, A-B). A positive PCR for leishmanias in the BM further confirmed the diagnosis of VL. Even though there was strong suspicion for leishmaniasis from day five (BM aspiration), the final diagnosis was established on day 19 and etiological treatment was initiated on day 23.

Hence, liposomal amphotericin B (light-sensitive drug) was applied at a daily dosage of 3 mg/kg for five days. An improvement in the laboratory data was observed from day 24 and the fever subsided on day 26 (platelets 72,000/μL, triglycerides 316 mg/dl, ferritin 3,948 ng/ml). On day 27, she was discharged from the hospital with oral methylprednisolone (32 mg daily). A complete recovery of the clinical manifestations and a vast improvement of the laboratory findings were finally seen on day 34 (ferritin 846 ng/ml, fibrinogen 182 mg/dl). Daily sessions of liposomal amphotericin B every 15 days for the next three months (maintenance therapy, six cycles totally) were applied. The patient remains afebrile and healthy with complete resolution of VL until today, at the age of 88.

## Discussion

Several molecular mechanisms for HLH have been proposed. The clinical presentation of the patient is not directly ascribed to VL pathogenesis and only molecular postulations can be made linking VL and HLH. An abnormal activation of monocytes, macrophages, dendritic cells, cytotoxic NK- or T-cells has been implicated [2]. An uncontrolled and ineffective immune response is the hallmark of HS [8]. More specifically, impaired perforin-dependent cytotoxicity, impaired control of infections, as well as dysregulated inflammasome activity affecting IL-1β and IL-18, are all examples of the cascade of abnormal function of the immune system in this syndrome [9]. Next generation sequencing has identified familial HLH genes, variants in primary immunodeficiency (PIDD)-associated genes or dysregulated immune activation or proliferation (DIAP) genes [10].

Zoonotic diseases are an important cause of HLH. Brucellosis, rickettsial diseases and Q fever are the most common bacterial etiologies. Regarding viral diseases, patients with avian influenza A subtype H5N1 and EBV infections are frequently implicated in the pathogenesis of HLH. Among the protozoa, most of the cases were reported in patients with VL. Regarding fungi, most of the cases were reported in HIV patients with histoplasmosis [11].

In the pandemic coronavirus disease 2019 (COVID-19) infection, the increased levels of inflammatory cytokines (hyperinflammation, cytokine storm syndrome) resemble the cytokine profile observed in cases of secondary HLH and MAS [12]. Severe hemophagocytosis on BM aspirates, cytopenia of two or more lineages and increased serum ferritin levels (≥ 2000 ng/mL) were shown in patients with severe COVID-19 [13]. The control of hypercytokinemia is the key to successfully treat secondary HLH/MAS. However, the effectiveness of cytokine blocking with anti-IL-1 and anti-IL-6 on secondary HLH/MAS has been limited [12,13].

VL is a rare cause of HS, linked to HLH. Several cases have been reported in the literature [14,15]. An unusual manifestation of VL mimicking lymphoma has also been described [16]. In most of these cases the diagnosis was delayed because the clinical suspicion for leishmaniasis as a trigger for HLH involves an experienced hematologist or hematopathologist in the BM morphology. The infusion of immunoglobulins did not improve the patient’s thrombocytopenia, because the latter was due to the infection. The delay for initiation of the etiological treatment was because of the pending BM biopsy, for the confirmation of the presence of the parasites in the BM, besides the aspiration. Various diagnostic assays for visceral leishmaniasis with diverse degrees of specificity and sensitivity have been proposed [16]; however, the gold standard remains the visual identification of the parasite in the BM. Nevertheless, due to the life cycle of the parasite, the presence of protozoa can be inconsistent between consecutive BM instances/images. The success rate of the initiation of amphotericin B is very high for treating VL [14].

In general, HS deriving from secondary HLH has a grave prognosis [17]. However, in recent years, the outcome of HLH has improved, due to a better molecular understanding of the syndrome, along with the initiation of intensive chemo- and immunotherapy. The prognosis of HLH in children differs by HLH subtype. In Japan, EBV-HLH is the most common HLH subtype [18]. Patients with relapsed or refractory disease after the application of the HLH-2004 protocol and those with the familial subtype should be treated with hematopoietic stem cell transplantation (HSCT) [17,18].

In cases of superficial interpretation of the diagnostic criteria for HLH, a secondary HLH due to malignancy or infection might incorrectly be considered as a primary HLH. Improper use of corticosteroids could cover up an underlying possible malignancy or infection and delay the initiation of the suitable treatment. A coexistence of both cancer and infection behind a secondary HLH should be excluded (EBV driver of lymphoma).

The specificity of the criteria for differential diagnosis between primary and secondary HLH is based on 2004 laboratory techniques and has not been extensively studied. These criteria are helpful in diagnosing the primary cause of HLH only in 20% of cases [19,20]. The optimal treatment of HLH remains unknown to this day. The inhibition of hyper-inflammation with immunosuppression is necessary; however this strategy might decrease the defense mechanisms against a possible primary infectious factor. Prolonged immunosuppression could lead to a reactivation of the initial trigger of HLH, thereby leading to a vicious cycle [8,19]. Clinicians should be cautious in distinguishing secondary HS from primary HS and should also define the underlying causes behind secondary HLH. VL must always be considered among the infectious etiologies resulting in HLH.

## Conclusions

We describe a case of HLH as a manifestation of VL. The clinical suspicion for leishmaniasis involves an experienced hematologist in the BM morphology. Due to the life cycle of the parasite, the presence of protozoa can be inconsistent between subsequent BM instances. In cases of superficial interpretation of the diagnostic criteria for HLH, a secondary HLH due to malignancy or infection might incorrectly be considered as a primary HLH. Thus, clinicians should be cautious in distinguishing secondary HS from primary HS.
